# Bartonella spp. isolated from wild and domestic ruminants in North America.

**DOI:** 10.3201/eid0603.000313

**Published:** 2000

**Authors:** C C Chang, B B Chomel, R W Kasten, R M Heller, H Ueno, K Yamamoto, V C Bleich, B M Pierce, B J Gonzales, P K Swift, W M Boyce, S S Jang, H J Boulouis, Y Piémont, G M Rossolini, M L Riccio, G Cornaglia, L Pagani, C Lagatolla, L Selan, R Fontana

**Affiliations:** School of Veterinary Medicine, University of California, Davis, California 95616, USA.

## Abstract

Bartonella species were isolated from 49% of 128 cattle from California and Oklahoma, 90% of 42 mule deer from California, and 15% of 100 elk from California and Oregon. Isolates from all 63 cattle, 14 deer, and 1 elk had the same polymerase chain reaction/restriction fragment length polymorphism profiles. Our findings indicate potential for inter- and intraspecies transmission among ruminants, as well as risk that these Bartonella spp. could act as zoonotic agents.


### *Bartonella* spp. Isolated from Wild and Domestic Ruminants in North America[1]

**Chao-chin Chang,* Bruno B. Chomel,* Rickie W. Kasten,* Remy Heller,† Katherine M. Kocan,‡ Hiroshi Ueno,§ Kazuhiro Yamamoto,* Vernon C. Bleich,¶ Becky M. Pierce,¶ Ben J. Gonzales,¶ Pamela K. Swift,¶ Walter M. Boyce,* Spencer S. Jang,* Henri-Jean Boulouis,# and Yves Piémont†**

*School of Veterinary Medicine, University of California, Davis, California, USA; †Institut de Bactériologie, Université Louis Pasteur, Strasbourg, France; ‡College of Veterinary Medicine, Oklahoma State University, Stillwater, Oklahoma, USA; §School of Veterinary Medicine, Rakuno-Gakuen University, Ebetsu, Hokkaido, Japan; ¶California Department of Fish and Game, Bishop, Rancho Cordova, California, USA; #Ecole Nationale Vétérinaire d'Alfort, 94704 Maisons-Alfort, France

***Bartonella*** species were isolated from 49% of 128 cattle from California and Oklahoma, 90% of 42 mule deer from California, and 15% of 100 elk from California and Oregon. Isolates from all 63 cattle, 14 deer, and 1 elk had the same polymerase chain reaction/restriction fragment length polymorphism profiles. Our findings indicate potential for inter- and intraspecies transmission among ruminants, as well as risk that these ***Bartonella*** spp. could act as zoonotic agents.

***Bartonella*** species have been identified as important zoonotic agents (1,2). Cats are the main reservoir of ***Bartonella henselae***, the agent that causes cat scratch disease in humans (1). Long-term bacteremia in cats and flea transmission from cat to cat, as confirmed by experimental infection, support a vectorborne transmission (3). Some human cases of cat scratch disease were not associated with any known exposure to cats (4), suggesting that other animal species may serve as reservoirs of ***Bartonella***. Recently, new ***Bartonella*** species have been isolated from a wide range of mammals, including rodents (5-10), lagomorphs (11), carnivores (12-14), and cervids (14,15). Similarly, 90% of 42 mule deer (***Odocoileus hemionus***) from California were bacteremic with ***Bartonella*** isolates that were similar to isolates from roe deer in France (15) by polymerase chain reaction/restriction fragment length polymorphism (PCR/RFLP) of the 16S rRNA and citrate synthase genes (14). Modes of transmission in these ruminants need to be established. Tick transmission has been suspected but not yet proven for dogs infected with ***B. vinsonii*** subsp. ***berkhoffii*** (16). Since fleas are less likely than ticks to infest cattle (17), ticks may play an important role in the transmission of ***Bartonella*** species from wild ruminants.

Our objectives were to determine if elk (***Cervus elaphus***), bighorn sheep (***Ovis canadensis***), and domestic cattle (***Bos taurus***) are infected with ***Bartonella*** and to determine the molecular relationships between ***Bartonella*** isolated from cattle and wild ruminants. We performed a cross-sectional study to compare the prevalence of ***Bartonella*** infection in a beef cattle herd in the California Sierra Nevada foothills and a dairy herd from the California Central Valley.

### The Study

In February 1997, 42 samples from free-ranging mule deer were obtained from the Round Valley population, Mono and Inyo counties, California. In November 1997, 84 samples were

Address for correspondence: Bruno B. Chomel, Department of Population Health and Reproduction, School of Veterinary Medicine, University of California, Davis, CA 95616, USA; fax: 530-752-2377; e-mail: bbchomel@ucdavis.edu.

[1]An earlier version of this paper was presented at the Second International Conference on Emerging Zoonoses, Strasbourg, France, November 5–9, 1998.

collected from bighorn sheep herds in California and New Mexico. During January and February 1998, 100 blood samples were collected from elk in California and Oregon. One hundred twenty-eight cattle samples were collected: 12 from Oklahoma beef cattle in April 1998 and 116 from two California herds from May to July 1998. Fifty-three samples were collected from a >4,000-head beef cattle herd in the Sierra Nevada foothills and 63 samples from a >1,500-head dairy herd in the Central Valley. These 116 cattle were all > 2 years of age. Blood samples collected into lysis-centrifugation tubes were plated within 48 hours. Blood samples collected into EDTA tubes were frozen at -70° until plated. Wildlife and domestic herds were selected on the basis of ongoing surveys by the California and Oregon Departments of Fish and Game and researchers at the Universities of California and Oklahoma.[2]

Blood samples were cultured on heart infusion agar containing 5% rabbit blood and incubated in 5% $CO_2$ at 35°C for at least 4 weeks (18). Gram staining and biochemical tests were performed on representative isolates, which were defined as isolates with a unique PCR/RFLP profile for each of the three ruminant species. Nine representative isolates were identified, including one cattle strain (pattern I), five deer strains (patterns I, II, IV, V, and VI), and three elk strains (patterns I, II, and III). Standard methods were used to test for various preformed enzymes and carbohydrate use. Preformed bacterial enzyme activity was tested by Microscan Rapid Anaerobe Panel (Dade International Inc., West Sacramento, CA) (19).

An approximately 400-bp fragment of the citrate synthase gene was amplified as described (20). The amplified product was digested with ***Taq***I and ***Hha***I and ***Mse***I restriction endonucleases and visualized by gel electrophoresis. Banding patterns were compared with ***B. henselae*** (strain U-4; University of California, Davis, CA).

Cellular fatty acid composition was analyzed for representative cattle, deer, and elk isolates. Isolates were grown on rabbit blood agar at 35°C for 5 days. Fatty acid methyl ester derivatives were separated on a Hewlett-Packard series II 5890 gas chromatograph.

The PCR products used for DNA sequencing were purified with Microcon centrifugal filter devices (Millipore Corp., Bedford, MA) and sequenced with a fluorescent-based automated sequencing system. Primer BhCS.1137n (5'-AATGCAAAAAGAACAGTAAACA-3') (20) was used for partial sequencing of the 400-bp product of the citrate synthase gene. Nine representative strains from ruminants and one ***B. henselae*** strain (strain U-4, University of California, Davis) were sequenced. The GAP program of GCG software (Wisconsin Sequence Analysis Package, Genetics Computer Group, version 10) was used for alignments and comparisons of sequences, based on the 276 bp of the citrate synthase gene.

Using Epi Info version 6.03, we performed a chi-square test to assess association between prevalence of bacteremia of ***Bartonella*** infection and herd location. The ***Bartonella*** infection prevalence ratio (PR) was calculated to show the proportionate increase of infection prevalence due to difference in herd location.

### Results

***Bartonella*** spp. were isolated from 5 (42%) of 12 Oklahoma cattle, 58 (50%) of 116 California cattle, 38 (90%) of 42 California mule deer, 15 (15%) of 100 elk, and none of 84 bighorn sheep. In the California beef cattle herd, 25 (96%) of 26 bulls and 22 (81%) of 27 cows were ***Bartonella*** bacteremic; in the dairy herd, 11 (17%) of 63 cows were bacteremic. ***Bartonella*** bacteremia prevalence in the Sierra Nevada foothills beef cattle herd was therefore significantly higher than in the Central Valley dairy cattle herd (PR = 5.1; 95% confidence interval [CI] = 2.9-8.8). Prevalence of ***Bartonella*** bacteremic cows in the foothills herd was also significantly higher (81% vs. 17%) than in the Central Valley dairy cattle herd (PR = 4.7; 95% CI = 2.7-8.2). For elk, bacteremia prevalence differed significantly ($p = 0.0002$) between California (0 of 47) and Oregon (15 [28%] of 53). No ***Bartonella***-bacteremic elk were found in the two California herds, but 11 (38%) of 29 elk from southwestern Oregon and 4 (17%) of 24 elk from northwestern Oregon were bacteremic.

The organisms isolated were short, slender gram-negative rods. By measuring preformed

[2]Collection sites for bighorn sheep were the Peninsular Ranges in California and the San Francisco River, Turkey Creek, and Red Rock in New Mexico. For elk, collection sites were the San Luis National Wildlife Refuge in Merced County and the Tupman Tule Elk State Reserve in Kern County (California); the Roseburg, Drain, and Demet herds, Douglas County (southwestern Oregon); and the Jewell Wildlife Area, Clatsop County (northwestern Oregon).

enzymes (Rapid Anaerobe Panel), the tested strains were found to be biochemically inert except for the production of peptidases, characteristic of the ***Bartonella*** profile (10077640).

Several strain profiles were observed by PCR/RFLP of the citrate synthase gene, using ***Taq***I and ***Hha***I and ***Mse***I endonucleases for deer (five profiles) and elk (three profiles) isolates (Figure). Conversely, all 63 cattle isolates had the same PCR/RFLP profile (Figure) with the same restriction enzymes. Overall, six different PCR/RFLP profiles were obtained from ***Bartonella*** isolated from cattle, deer, and elk. ***Bartonella*** isolated from cattle (63 of 63 tested; lanes 2, 12, and 22), mule deer (14 of 38 tested; lanes 3, 13, and 23), and an elk from southwestern Oregon (1 of 11 tested; lanes 10, 20, and 30) yielded the same PCR/RFLP profile (pattern I) with the three enzymes used. A second profile (pattern II) was obtained for ***Bartonella*** isolated from elk captured in northwestern Oregon (4 of 4 tested; lanes 8, 18, and 28) and from mule deer (5 of 38 tested; lanes 4, 14, and 24). A third profile (pattern III) was obtained for 10 of the 11 ***Bartonella*** isolated from elk captured in southwestern Oregon (lanes 9, 19, and 29). The other three profiles (patterns IV, V, and VI) were obtained for ***Bartonella*** isolated from mule deer ([pattern IV: 12 of 38 tested; lanes 6, 16, and 26]; [pattern V: 5 of 38 tested; lanes 5, 15, and 25]; and [pattern VI: 2 of 38 tested; lanes 7, 17, and 27]).

The cellular fatty acid composition was characteristic of the ***Bartonella*** genus for all isolates. The main fatty acids observed for the cattle, deer, and elk strains were octadecanoic acid ($C_{18:1}$, 45%-66%), octadecanoic acid ($C_{18:0}$, 12%-23%), and hexadecanoic acid ($C_{16:0}$, 13%-20%).

After pairwise comparisons, the partial sequencing analysis (276 bp) of the citrate synthase gene for the nine representative ruminant strains showed a high percentage of DNA similarity, from 93.12% to 100% (Table 1). The strains cattle-1, deer-1, and elk-1 belonging to the PCR/RFLP pattern I had 95.65% to 99.64% DNA similarity. The strains deer-2 and elk-2 with PCR/RFLP pattern II had 100% DNA similarity. The strain deer-1 with PCR/RFLP pattern I was closely related (98.91% DNA identity) to the strain deer-2 with PCR/RFLP pattern II. For strains deer-4 and deer-5, corresponding to PCR/RFLP patterns IV and V (similar digestion profiles with ***HhaI*** and ***MseI*** endonucleases and different profiles from ***TaqI*** endonuclease), a 98.55% DNA similarity was observed. Partial sequence analysis (276 bp) of the citrate synthase gene showed that all strains from ruminants were closely related to ***B. weissii***, a ***Bartonella*** species isolated from domestic cats (Table 2).

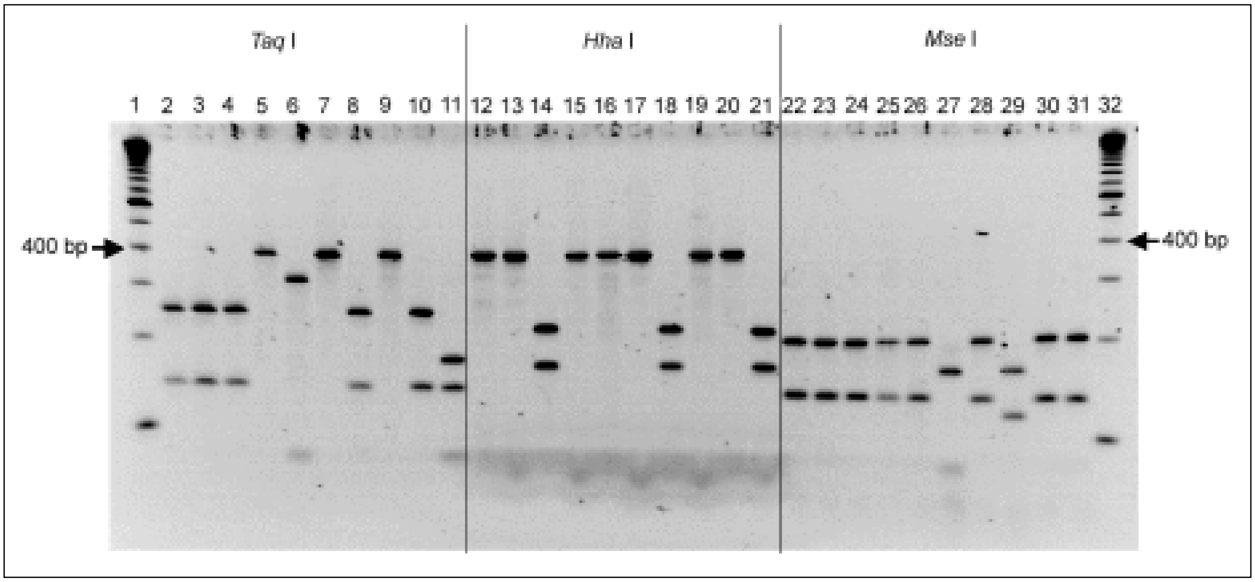

Figure. Polymerase chain reaction/restriction fragment length polymorphism of the citrate synthase gene of isolates from cattle, deer, and elk, with ***Taq***I, ***Hha***I, and ***Mse***I endonucleases. Lanes 1 and 32, standard 100-bp molecular ladder; lanes 2, 12, and 22, cattle isolate; lanes 3 to 7, 13 to 17, and 23 to 27, deer isolates; lanes 8 to 10, 18 to 20, and 28 to 30, elk isolates; lanes 11, 21, and 31, ***B. henselae*** strain.

Table 1. DNA similarity values and GenBank accession numbers based on 276 bp of the citrate synthase gene of the nine representative ruminant strains

| Organism/ | % Similarity by strain | | | | | | | | |
|---|---|---|---|---|---|---|---|---|---|
| accession no. | Cattle-1 | Deer-1 | Deer-2 | Deer-4 | Deer-5 | Deer-6 | Elk-1 | Elk-2 | Elk-3 |
| Cattle-1 AF228768 | 100.00 | 95.65 | 96.01 | 94.57 | 94.57 | 94.57 | 99.64 | 96.01 | 94.57 |
| Deer-1 AF228769 | - | 100.00 | 98.91 | 93.84 | 93.84 | 93.12 | 95.65 | 98.91 | 93.84 |
| Deer-2 AF228771 | - | - | 100.00 | 94.20 | 94.20 | 93.48 | 96.01 | 100.00 | 94.20 |
| Deer-4 AF228774 | - | - | - | 100.00 | 98.55 | 94.93 | 94.57 | 94.20 | 94.57 |
| Deer-5 AF228775 | - | - | - | - | 100.00 | 94.20 | 94.57 | 94.20 | 94.57 |
| Deer-6 AF228776 | - | - | - | - | - | 100.00 | 94.20 | 93.48 | 96.01 |
| Elk-1 AF228770 | - | - | - | - | - | - | 100.00 | 96.01 | 94.57 |
| Elk-2 AF228772 | - | - | - | - | - | - | - | 100.00 | 94.20 |
| Elk-3 AF228773 | - | - | - | - | - | - | - | - | 100.00 |

Table 2. DNA similarity values based on 276 bp of the citrate synthase gene of the nine ruminant strains compared with those of the ***Bartonella*** strains in GenBank

| | % Similarity by strain | | | | | | | | |
|---|---|---|---|---|---|---|---|---|---|
| Organism/accession no. | Cattle-1 | Deer-1 | Deer-2 | Deer-4 | Deer-5 | Deer-6 | Elk-1 | Elk-2 | Elk-3 |
| ***B. bacilliformis*** U28076 | 86.59 | 87.68 | 87.32 | 84.78 | 85.51 | 84.78 | 86.59 | 87.32 | 87.68 |
| ***B. grahamii*** Z70016 | 90.22 | 90.22 | 90.58 | 91.67 | 90.22 | 90.58 | 90.58 | 90.58 | 89.49 |
| ***B. taylorii*** Z70013 | 88.41 | 87.32 | 86.96 | 87.68 | 87.68 | 87.68 | 88.04 | 86.96 | 88.04 |
| ***B. tribocorum*** AJ005494 | 89.86 | 89.13 | 89.49 | 90.58 | 89.13 | 88.41 | 89.49 | 89.49 | 88.04 |
| ***B. doshiae*** Z70017 | 88.41 | 86.59 | 86.96 | 86.96 | 86.23 | 87.68 | 88.04 | 86.96 | 85.87 |
| ***B. vinsonii*** subsp. ***vinsonii*** U28074 | 88.69 | 89.05 | 87.96 | 88.69 | 88.69 | 89.42 | 88.32 | 87.96 | 87.96 |
| ***B. vinsonii*** subsp. ***berkhoffii*** U28075 | 89.86 | 89.49 | 89.13 | 87.68 | 87.68 | 88.41 | 89.49 | 89.13 | 86.96 |
| ***B. vinsonii*** subsp. ***arupensis*** U77057 | 90.22 | 89.13 | 89.13 | 90.94 | 90.94 | 90.22 | 89.86 | 89.13 | 88.77 |
| ***B. weissii*** AF071190 | 99.64 | 95.65 | 96.01 | 94.57 | 94.57 | 94.20 | 100.00 | 96.01 | 94.57 |
| ***B. clarridgeiae*** U84386 | 90.58 | 89.49 | 89.86 | 88.77 | 88.04 | 88.77 | 90.22 | 89.86 | 89.49 |
| ***B. henselae*** strain U-4 | 90.58 | 88.41 | 89.49 | 88.77 | 88.77 | 87.32 | 90.22 | 89.49 | 87.68 |
| ***B. henselae*** strain Houston-1 L38987 | 90.58 | 88.41 | 89.49 | 88.77 | 88.77 | 87.32 | 90.22 | 89.49 | 87.68 |
| ***B. koehlerae*** AF176091 | 89.13 | 88.41 | 88.77 | 89.49 | 88.77 | 87.32 | 88.77 | 88.77 | 87.32 |
| ***B. quintana*** Z70014 | 90.22 | 88.04 | 88.41 | 87.68 | 86.96 | 88.41 | 89.86 | 88.41 | 88.77 |
| ***B. elizabethae*** Z70009 | 88.41 | 88.04 | 88.41 | 90.22 | 88.77 | 89.49 | 88.04 | 88.41 | 88.04 |
| Strain C7-rat Z70020 | 88.41 | 88.04 | 88.41 | 90.22 | 88.77 | 89.49 | 88.04 | 88.41 | 88.04 |
| Strain C5-rat Z70018 | 88.77 | 88.77 | 89.13 | 90.22 | 88.77 | 87.32 | 88.77 | 89.13 | 87.68 |
| Strain C4-phy Z70019 | 87.32 | 86.23 | 86.96 | 86.96 | 86.96 | 86.59 | 86.96 | 86.96 | 85.15 |
| Strain C1-phy Z70022 | 86.59 | 85.51 | 86.23 | 86.23 | 86.23 | 85.87 | 86.23 | 86.23 | 84.42 |
| Strain R-phy2 Z70011 | 87.32 | 86.23 | 86.96 | 86.96 | 86.96 | 86.59 | 86.96 | 86.96 | 85.15 |
| Strain R-phy1 Z70010 | 88.04 | 87.68 | 88.04 | 87.32 | 87.32 | 87.32 | 87.68 | 88.04 | 85.87 |
| Strain N40 Z70012 | 90.22 | 88.77 | 89.13 | 88.77 | 87.32 | 87.32 | 89.86 | 89.13 | 86.96 |
| Strain A1 U84372 | 88.77 | 87.68 | 88.77 | 88.04 | 87.32 | 88.04 | 88.41 | 88.77 | 86.23 |
| Strain A2 U84373 | 88.41 | 87.32 | 88.41 | 87.68 | 87.68 | 87.68 | 88.04 | 88.41 | 86.23 |
| Strain A3 U84374 | 88.77 | 88.04 | 88.77 | 88.04 | 88.04 | 88.04 | 88.41 | 88.77 | 86.23 |
| Strain B1 U84375 | 88.49 | 89.13 | 88.77 | 88.77 | 88.77 | 89.49 | 89.13 | 88.77 | 88.04 |
| Strain B2 U84376 | 89.86 | 89.49 | 89.13 | 88.41 | 88.41 | 89.13 | 89.49 | 89.13 | 87.68 |
| Strain C1 U84377 | 88.77 | 89.13 | 88.77 | 87.68 | 86.96 | 88.41 | 88.41 | 88.77 | 86.59 |
| Strain C2 U84378 | 88.77 | 89.13 | 88.77 | 87.68 | 86.96 | 88.41 | 88.41 | 88.77 | 86.59 |
| Strain D1 U84379 | 89.86 | 88.77 | 88.77 | 90.58 | 90.58 | 89.86 | 89.49 | 88.77 | 88.41 |
| Strain D2 U84380 | 90.22 | 89.13 | 89.13 | 90.94 | 90.94 | 90.22 | 89.86 | 89.13 | 88.41 |
| Strain D3 U84381 | 90.58 | 89.86 | 89.49 | 90.58 | 90.58 | 90.58 | 90.22 | 89.49 | 88.77 |
| Strain D4 U84382 | 90.22 | 89.13 | 89.13 | 90.94 | 90.94 | 90.22 | 89.86 | 89.13 | 88.77 |
| Strain D5 U84383 | 89.49 | 89.13 | 88.41 | 90.22 | 90.22 | 89.49 | 89.13 | 88.41 | 88.04 |
| Strain D6 U84384 | 90.58 | 89.86 | 89.49 | 90.58 | 90.58 | 90.58 | 90.22 | 89.49 | 88.77 |
| Strain D7 U84385 | 90.22 | 89.13 | 89.13 | 90.94 | 90.94 | 90.22 | 89.86 | 89.13 | 88.41 |

### Conclusion

This is the first published report of isolation of ***Bartonella*** spp. from free-ranging wild ruminants and domestic ruminants in North America. Our results suggest that deer, elk, and domestic cattle are possible reservoirs of ***Bartonella*** spp. Selected bighorn sheep populations from California and New Mexico appeared to be free of ***Bartonella***. The first report of infection of cattle with a ***Bartonella*** organism was made in 1934 by Donatien and Lestoquard, who proposed the name ***B. bovis*** or ***Haemobartonella bovis*** (21). In 1942, Lotze and Yiengst also described ***Bartonella***-like structures in American cattle (22); however, their identifications of ***Bartonella***-like structures were based only on the morphologic aspects of these organisms in red blood cells also infected with ***Theileria*** or ***Anaplasma***, two well-known tickborne infections.

Partial sequencing analysis of the citrate synthase gene of the ruminant strains showed that they were all closely related to each other and to a feline strain, ***B. weissii***. Further studies by DNA-DNA hybridization may determine if these strains are specific to ruminants but closely related to ***B. weissii***, or if they are in fact ***B. weissii***. If the ruminant strains are identical to ***B. weissii***, the high prevalence (89%) of ***Bartonella*** bacteremia observed in beef cattle may indicate that ruminants are the main reservoirs of ***B. weissii***, which is not commonly isolated from cats.

The prevalence of ***Bartonella*** bacteremia was high in beef cattle and mule deer, possibly related to exposure to potential vectors. Since fleas are rarely observed on cattle and tick infestation is common in both cattle and deer, ticks are a possible source of infection for ruminants (17). Furthermore, ***Bartonella*** DNA has recently been demonstrated in a high percentage of ticks infesting roe deer in Europe (23,24). The herd of beef cattle from the Sierra Nevada foothills, where tick infestation is common, has permanent access to open pastures. In contrast, the dairy cattle herd from the Central Valley has little or no access to pastures and tick infestations are not commonly observed (R. BonDurant, pers. comm.). Therefore, geographic differences in the prevalence of ***Bartonella*** infection in California cattle herds warrant further investigation for possible tick transmission of ***Bartonella*** spp. among these animals.

PCR/RFLP analysis of the citrate synthase gene has been widely used for identification of ***Bartonella*** organisms to the species level (25-27). We identified one PCR/RFLP profile for all the cattle isolates, but several profiles for deer and elk. This diversity by geographic location is of epidemiologic interest and warrants further investigation. Only one elk from southwestern Oregon had a strain with a similar PCR/RFLP profile to that of domestic cattle, suggesting that wild ruminants could be infected with ***Bartonella*** species that are not commonly shared with cattle.

Our findings also suggest that transmission of ***Bartonella*** may occur among cattle and wildlife, especially mule deer, which are more abundant in the western USA than elk and are more likely to be sympatric with cattle. Collection and analysis of ticks on wild animals and cattle and from the environment will be necessary to determine if ticks can be infected with ***Bartonella*** species. Whether ***Bartonella*** isolated from these ruminants are human pathogens is still unclear. The recent report of a cattle rancher who was infected with a new ***B. vinsonii*** subspecies (28) warrants further investigation to establish if these ***Bartonella*** species could be zoonotic and whether humans could potentially be infected by tick bites during work or recreation.

Dr. Chang is pursuing his Ph.D. in epidemiology at the University of California, Davis, under the direction of Bruno B. Chomel. His research interests include epidemiology of zoonoses, especially the molecular epidemiology of ***Bartonella*** infections and potential vectors for ***Bartonella*** spp. transmission.

Dr. Chang's research was funded by a grant from the Center for Companion Animal Health, University of California, Davis, California, USA.

### References

1. Bass JW, Vincent JM, Person DA. The expanding spectrum of ***Bartonella*** infections. II. Cat scratch disease. Pediatr Infect Dis J 1997;16:163-79.
2. Chomel BB. Cat-scratch disease and bacillary angiomatosis. Rev Sci Tech 1996;15:1061-73.
3. Chomel BB, Kasten RW, Floyd-Hawkins KA, Chi B, Yamamoto K, Roberts-Wilson J, et al. Experimental transmission of ***Bartonella henselae*** by the cat flea. J Clin Microbiol 1996;34:1952-6.
4. Margileth AM. Cat-scratch disease. A therapeutic dilemma. Vet Clin North Am Small Anim Pract 1987;17:91-103.

5. Birtles RJ, Fichet-Calvet E, Raoult D, Ashford RW. Detection and genotypic differentiation of ***Bartonella*** species infecting a Tunisian ***Psammomys obesus*** population. 13th Sesqui-annual meeting of the American Society of Rickettsiology; 1997 Sep 21-24; Seven Springs Mountain Resort, Champion, Pennsylvania. [Abstract 34].
6. Ellis BA, Regnery RL, Beati L, Bacellar F, Rood M, Glass GG, et al. Rats of the genus ***Rattus*** are reservoir hosts for pathogenic ***Bartonella*** species: an Old World origin for a New World disease? J Infect Dis 1999;180:220-4.
7. Heller R, Kubina M, Delacour G, Mahoudeau I, Lamarque F, Artois M, et al. Prevalence of ***Bartonella*** spp. in blood of wild small rodents. Abstracts of the General Meeting of the American Society for Microbiology, 1997;97:115.
8. Heller R, Riegel P, Hansmann Y, Delacour G, Bermond D, Dehio C, et al. ***Bartonella tribocorum*** sp. nov., a new ***Bartonella*** species isolated from the blood of wild rats. Int J Syst Bacteriol 1998;48:1333-9.
9. Hofmeister EK, Kolbert CP, Abdulkarim AS, Magera JMH, Hopkins MK, Uhl JR, Ambyaye A, Telford III SR, Cockerill III FR, Persing DH. Cosegregation of a novel ***Bartonella*** species with ***Borrelia burgdorferi*** and ***Babesia microti*** in ***Peromyscus leucopus.*** J Infect Dis 1998;177:409-16.
10. Kosoy MY, Regnery RL, Tzianabos T, Marston EL, Jones DC, Green D, et al. Distribution, diversity, and host specificity of ***Bartonella*** in rodents from the southeastern United States. Am J Trop Med Hyg 1997;57:578-88.
11. Heller R, Kubina M, Mariet P, Riegel P, Delacour G, Dehio C, Lamarque F, Kasten R, Boulouis HJ, Monteil H. ***Bartonella alsatica*** sp. nov., a new ***Bartonella*** species isolated from the blood of wild rabbits. Int J Syst Bacteriol 1999;49:283-8.
12. Kordick DL, Swaminathan B, Greene CE, Wilson KH, Whitney AM, O'Connor S, et al. ***Bartonella vinsonii*** subsp. ***berkhoffii*** subsp. nov., isolated from dogs; ***Bartonella vinsonii*** subsp. ***vinsonii***; and emended description of ***Bartonella vinsonii***. Int J Syst Bacteriol 1996;46:704-9.
13. Kelly PJ, Rooney JJA, Marston EL, Jones DC, Regnery RL. ***Bartonella henselae*** isolated from cats in Zimbabwe [letter]. Lancet 1998;351:1706.
14. Chomel BB, Kasten RW, Chang CC, Yamamoto K, Heller R, Maruyama S, et al. Isolation of ***Bartonella*** spp. from California wildlife. International Conference on Emerging Infectious Diseases; 1998 Mar 8-11; Atlanta, Georgia. p. 21.10.
15. Heller R, Kubina M, Delacour G, Lamarque F, Van Laere G, Kasten R, et al. Isolation of ***Bartonella*** spp. from wildlife in France. International Conference on Emerging Infectious Diseases; 1998 Mar 8-11; Atlanta, Georgia. p. 21.18.
16. Pappalardo BL, Correa MT, York CC, Peat CY, Breitschwerdt EB. Epidemiologic evaluation of the risk factors associated with exposure and seroreactivity to ***Bartonella vinsonii*** in dogs. Am J Vet Res 1997;58:467-71.
17. Wall R, Shearer D. Veterinary entomology. 1st ed. London: Chapman and Hall; 1997. p. 338.
18. Chomel BB, Abbott RC, Kasten RW, Flowd-Hawkins KA, Kass PH, Glaser CA, et al. ***Bartonella henselae*** prevalence in domestic cats in California: risk factors and association between bacteremia and antibody titers. J Clin Microbiol 1995;33:2445-50.
19. Welch DF, Hensel DM, Pickett DA, San Joaquin VH, Robinson A, Slater LN. Bacteremia due to ***Rochalimaea henselae*** in a child: practical identification of isolates in the clinical laboratory. J Clin Microbiol 1993;31:2381-6.
20. Norman AF, Regnery R, Jameson P, Greene C, Krause DC. Differentiation of ***Bartonella***-like isolates at the species level by PCR-restriction fragment length polymorphism in the citrate synthase gene. J Clin Microbiol 1995;33:1797-803.
21. Donatien A, Lestoquard F. Sur une ***Bartonella*** nouvelle du boeuf, ***Bartonella bovis*** n. sp. Bull Soc Pathol Exot 1934;7:652-4.
22. Lotze JC, Yiengst MJ. Studies on the nature of ***Anaplasma***. Am J Vet Res 1942;3:312-20.
23. Bergmans AMC. Cat scratch disease: Studies on diagnosis and identification of reservoirs and vectors. Ph.D. Thesis, Utrecht University, The Netherlands, 1996; 152pp.
24. Schouls LM, Van de Pol I, Rijpkema SG, Schot CS. Detection and identification of ***Ehrlichia***, ***Borrelia burgdorferi*** sensu lato, and ***Bartonella*** species in Dutch ***Ixodes ricinus*** ticks. J Clin Microbiol 1999;37:2215-22.
25. Koehler JE, Quinn FD, Berger TG, LeBoit PE, Tappero JW. Isolation of ***Rochalimaea*** species from cutaneous and osseous lesions of bacillary angiomatosis. N Engl J Med 1992;327:1625-31.
26. Regnery RL, Andersen BE, Clarridge JE III, Rodriguez-Barradas MC, Jones DC, Carr JH. Characterization of a novel ***Rochalimaea*** species, ***R. henselae*** sp. nov., isolated from blood of a febrile, human immunodeficiency virus-positive patient. J Clin Microbiol 1992;30:265-74.
27. Dolan MJ, Wong MT, Regnery RL, Jorgensen JH, Garcia M, Peters J, et al. Syndrome of ***Rochalimaea henselae*** adenitis suggesting cat scratch disease. Ann Intern Med 1993;118:331-6.
28. Welch DF, Carroll KC, Hofmeister EK, Persing DH, Robison DA, Steigerwalt AG, et al. Isolation of a new subspecies, ***Bartonella vinsonii*** subsp. ***arupensis***, from a cattle rancher: identity with isolates found in conjunction with ***Borrelia burgdorferi*** and ***Babesia microti*** among naturally infected mice. J Clin Microbiol 1999;37:2598-601.
